# Supplementary material for: Splice-Junction-Based Mapping of Alternative Isoforms in the Human Proteome
Source: Cell Rep. Author manuscript; Available in PMC 2020 Jan 15. (PMC6961840; doi:10.1016/j.celrep.2019.11.026)

A

sp|Q8WZ42|TITIN_HUMAN|ENSG00000155657|MXE1|1073|chr2|178642317|178650834|−2|r36|T1,sp|Q8WZ42|TITIN_
KPEAPPAKVPEVPKK q value: 0.0023869 Tr_novel:TRUE RefSeq_Novel:TRUE
Search result spec prec mz: 404.9993 Actual spec prec mz: 404.99929
Fragments matched per AA: 1.67 Proportion of top 20 peaks matched: 0.25

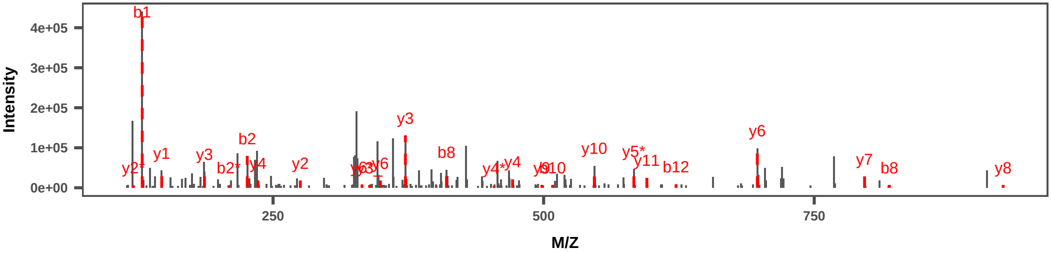


B

Scatterplot of predicted elution time
Fitting R2: 0.775
Novel peptide residual Z score: 0.785
Number of peptides: 2597

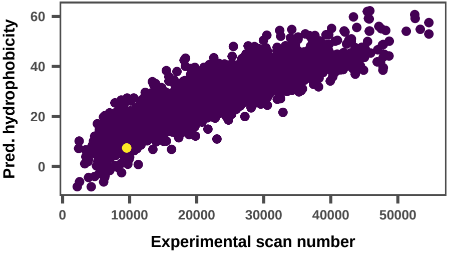


C

Distributions of residuals from best−fit line
of predicted RT vs Expt. scan number
Line: Z score of novel peptide
Z: 0.785

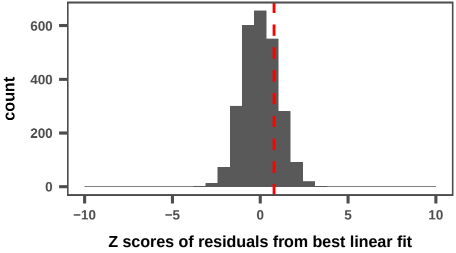

Supplement: 2 [file NIHMS1546469-supplement-2.zip › DF1/PXD010154/Heart/Heart_75_TTN_KPEAPPAKVPEVPKK.pdf]
